# Extrapleural pneumonectomy for sarcoma: Outcomes of adult patients at a specialized center

**DOI:** 10.1002/cnr2.2065

**Published:** 2024-04-16

**Authors:** Betty Y. Zhang, Ashley R. Wilson‐Smith, Elizabeth A. Connolly, Madeleine C. Strach, Nathan Ussher, Tristan Yan, Vivek A. Bhadri

**Affiliations:** ^1^ Department of Medical Oncology Chris O'Brien Lifehouse Sydney New South Wales Australia; ^2^ Faculty of Medicine and Health The University of Sydney Sydney New South Wales Australia; ^3^ Department of Cardiothoracic Surgery Royal Prince Alfred Hospital Sydney New South Wales Australia

**Keywords:** cardiothoracic surgery, complications, recurrence, sarcoma, survival

## Abstract

**Background:**

Extrapleural pneumonectomy (EPP) is a complex surgical procedure involving en‐bloc resection of the parietal and visceral pleura, lung, pericardium, and ipsilateral diaphragm. Small case series of pleural‐based sarcoma of predominantly pediatric patients suggest EPP may be a life‐prolonging surgical option. We aimed to describe the characteristics and outcomes of adults who underwent EPP at a specialized sarcoma center.

**Methods:**

Clinicopathologic variables, surgical details, and follow‐up information were extracted for patients undergoing EPP for pleural‐based sarcoma between August 2017 and December 2020. Primary outcomes were event‐free survival (EFS) and overall survival (OS) from the date of EPP. Secondary outcomes were disease‐free interval (DFI) prior to EPP, and early and late postoperative complications.

**Results:**

Eight patients were identified, seven with soft tissue sarcoma and one with bone sarcoma. Patients had either localized disease with a primary thoracic sarcoma, sarcoma recurrent to the thorax, or de novo metastatic disease. All patients underwent resection of their pleural‐based sarcoma by an experienced cardiothoracic surgeon, and some patients had pre or postoperative treatment. The perioperative morbidity was comparable with previously published reports of EPP performed in mesothelioma patients. At median follow‐up of 22.5 months, median EFS was 6.0 months and OS was 20.7 months. Six patients (75%) had disease recurrence; five (62.5%) died of progressive disease. Two patients (25%) had not recurred: one died of a radiation‐related esophageal rupture, and one was alive with no evidence of disease at 37.0 months. Characteristics of those with the longest EFS included low‐grade histology and achieving a metabolic response to preoperative chemotherapy.

**Conclusions:**

In adults with pleural‐based sarcoma, EPP is rarely curative but appears to be a feasible salvage procedure when performed at specialized centers. Patient selection is critical with strong consideration given to multimodal therapy to optimize patient outcomes. In the absence of a confirmed response to neoadjuvant treatment, long term survival is poor and EPP should not be recommended.

## 1 INTRODUCTION

Sarcomas in adults are rare, with an incidence of less than six per 100 000 per year^1^ and primary pulmonary sarcomas are estimated to account for 0.4%–1.1% of all thoracic malignancies.^2^ Patients with pleural‐based sarcomas, whether found at initial diagnosis or disease recurrence, are challenging to manage and have a poor prognosis with 5‐year overall survival (OS) of 43%–60%.^2^, ^3^ A radical operation, such as extrapleural pneumonectomy (EPP), can be considered as part of a multimodal therapeutic approach in conjunction with chemotherapy and/or radiotherapy (RT) to obtain long‐term disease control in such patients.^4^ EPP is a complex procedure and consists of en‐bloc resection of the parietal and visceral pleura, lung, pericardium, and ipsilateral diaphragm.^4^ First described in 1949 for the management of pulmonary tuberculosis, experience with EPP has been chiefly gained in the management of pleural mesothelioma and more recently, for select patients with thymomas, advanced non‐small cell lung cancers and sarcomas.^3^, ^5^, ^6^ Irrespective of the tumor type, EPP should always be performed by an experienced cardiothoracic team in a high‐volume thoracic surgery center due to the potential for high morbidity.^7^ Given the rarity of pleural‐based sarcoma, literature on outcomes following EPP is sparse and no prospective studies exist. Small, predominantly pediatric case series have reported the outcomes of EPP for the management of pleural‐based sarcoma either as treatment for the primary disease or as salvage treatment of sarcoma recurrent to the pleura. Authors of two recently published case series suggest that EPP is a feasible and life‐prolonging procedure in select patients in combination with chemotherapy and radiation.^7^, ^8^ For this study, we aimed to determine the characteristics and disease outcomes of adult patients who underwent EPP at a tertiary, high‐volume cardiothoracic center co‐located with a specialized sarcoma center in Sydney, Australia.

## 2 METHODS

We report a case series identified from a retrospective chart review of all patients with pleural‐based sarcoma who underwent EPP between August 2017 and December 2020 at the Chris O'Brien Lifehouse/Royal Prince Alfred Hospital sarcoma unit. Patients were identified by review of a prospective database.

Data were collected with the approval of the local institutional review board and waiver of informed consent due to the retrospective nature of this study (HREC #2019/ETH11837).

All patients were discussed at multidisciplinary tumor board. The decision to administer perioperative systemic therapy was individualized and based on tumor chemosensitivity, prior treatment received, patient fitness, and preference. Systemic therapy was more likely to be delivered if the patient was fit and the tumor was a chemo‐sensitive subtype. The decision to deliver radiation was based on radiosensitivity, margin status and tumor grade.

Patient demographics, histological subtype, treatment and outcome details, and treatment complications were extracted from electronic medical records. The primary outcomes were event‐free survival (EFS) and overall survival (OS). EFS was defined as the interval between the date of EPP and the date of the first event, defined as disease recurrence (local or metastatic) or death. OS was defined as the interval from EPP until death from any cause. Patients were censored at the date of the last follow‐up. Secondary outcomes were disease‐free interval (DFI) prior to EPP and early (≤30 days) and late (>30 days) postoperative complications. In patients who underwent EPP for recurrent disease, DFI was defined as the interval from the completion of treatment for the last incidence of disease to EPP. Descriptive epidemiological methods were used to illustrate the demographics, tumor characteristics, and treatment patterns of the cohort. Survival probabilities were calculated using the Kaplan–Meier method.

## 3 RESULTS

### 3.1 Demographics and disease characteristics

Eight patients were identified; the median age was 41 years (range 22–70 years) at the time of EPP. Baseline patient demographics and disease characteristics are presented in Table 1. Seven patients had soft tissue sarcoma (three of synovial sarcoma, one each of intimal sarcoma, malignant solitary fibrous tumor, rhabdomyosarcoma transformed from non‐seminomatous germ cell tumor, and undifferentiated pleomorphic sarcoma) and one patient had bone sarcoma (chondrosarcoma, grade 1–2). Disease status included three patients with localized disease (primary sarcoma of the thorax), one with de novo pulmonary sarcoma with a solitary brain metastasis, and four with recurrent sarcoma with metastatic disease involving the thorax. All EPP procedures were performed by a cardiothoracic surgeon with extensive experience in complex thoracic oncology. All patients were informed that surgery was considered by the MDT to be the only curative intent treatment for their pleural‐based sarcoma and that without surgery, all other treatments would be palliative in nature.

**TABLE 1:** Patient demographics and disease characteristics.

Patient	Age (years)	Diagnosis	Disease status	Number of recurrences (DFI)	Preoperative treatment	Postoperative treatment	Type of recurrence	EFS from EPP (months)	OS from EPP (months)	Status at last follow up
1	70	Malignant solitary fibrous tumor	Primary	—	Nil	Adjuvant RT 56 Gy/28#	Distant (intra‐abdominal)	16.4	20.7	Died of disease
2	64	Intimal sarcoma	Primary	—	Nil	Nil^a^	Distant (brain)	2.0	2.4	Died of disease
3	22	Synovial sarcoma	Recurrent	2 (DFI 6.3 months)	Progressed on immunotherapy clinical trial within 1 month before surgery	Nil	Distant (T10‐12 epidural space with cord compression)	2.7	8.3	Died of disease
4	36	Synovial sarcoma	Primary	—	Neoadjuvant AI x 3	Adjuvant RT 60 Gy/30#	Nil	42.3 (censored)	42.3	Died of RT complication (esophageal perforation)
5	43	Synovial sarcoma	Recurrent	2 (DFI 9.7 months)	Nil^b^	Nil	Local recurrence (ipsilateral pleura)	6.0	26.2	Died of disease
6	38	Rhabdomyosarcoma (transformed from non‐seminomatous germ cell tumor)	Recurrent	1 (DFI 2.3 months)	Nil	Adjuvant VAC x 3	Distant (contralateral lung)	3.2	12.5	Died of disease
7	44	Chondrosarcoma, grade 1–2	Recurrent	5 (DFI 8.4 months)	Nil	Nil	Nil	37.0 (censored)	37.0 (censored)	No evidence of disease
8	26	Undifferentiated pleomorphic sarcoma	De novo metastatic thoracic tumor	—	Neoadjuvant AI x 4	Nil	Local recurrence (right pulmonary artery)	20.7	23.0 (censored)	Alive with disease—palliative GD

Abbreviations: AI, doxorubicin/ifosfamide; DFI, disease‐free interval; EFS, event‐free survival; GD, gemcitabine/docetaxel; Gy, Gray; OS, overall survival; RT, radiation; VAC, vincristine/actinomycin/cyclophosphamide.

^a^ Planned for adjuvant radiation but progressed and died rapidly.

^b^ Nil adjuvant chemotherapy due to the receipt of adjuvant doxorubicin/ifosfamide x 3 for primary tumor treatment and neoadjuvant doxorubicin/ifosfamide x 3 for treatment of first recurrence.

### 3.2 Surgical treatment, morbidity, and mortality

EPP was performed in all patients with the aim of complete cytoreductive surgery of all visible tumor, the exact surgical approach was determined by the location of the tumor, involvement of surrounding structures and the health status of the patient. Surgical characteristics and postoperative complications are presented in Table 2. Tumor diameter ranged from single lesions measuring 90–250 mm (occupation of the entire superior mediastinum). Five patients had complicated involvement of local structures, necessitating meticulous dissection. Six patients had clear surgical margins on review of their histopathology. No deaths occurred within 30 days of EPP. Three patients developed early postoperative complications; one developed ventilator‐associated pneumonia with new‐onset atrial fibrillation with rapid ventricular response requiring transesophageal cardioversion, one developed an ipsilateral hydropneumothorax requiring chest tube insertion, and one developed an ipsilateral hydropneumothorax that did not require chest tube insertion. One patient developed severe late postoperative complications with group A streptococcal (GAS) bacteremia, with seeding to the mitral valve and resultant septic cardiomyopathic shock. All patients required planned postoperative intensive care support, with subsequent discharge to a specialized thoracic ward for routine postoperative care. The median operation time was 4 hours (range 2–6), and the median hospital length of stay was 14 days (range 9–45).

**TABLE 2:** Surgical characteristics, early, and late postoperative complications.

Patient	Age (years)	Complex involvement of local structures	Early Complications	Late complications	Margin status
1	70	Tumor tamponade, pericardial and diaphragmatic involvement and invasion	Nil	Nil	R0
2	64	Extension to right superior pulmonary vein, right upper/middle lobe collapse, atrial tumor thrombus	Ventilator‐associated pneumonia; AF with RVR requiring TOE cardioversion	Nil	R1
3	22	Nil		Nil	R0
4	36	Proximal aortic involvement Compression of left main bronchus No plane between the lesion and aortic wall	Nil	GAS bacteremia, infective endocarditis Esophageal and aortic reconstruction Repeated space washouts Candidemia	R0
5	43	Right costophrenic angle	Ipsilateral hydropneumothorax	Nil	R0
6	38	Mediastinal displacement, left common carotid	Nil	Nil	Not reported
7	44	Nil	Nil	Nil	R0
8	26	Nil	Ipsilateral hydropneumothorax	Nil	R0

Abbreviations: AF, atrial fibrillation; GAS, group A streptococcus; R0, microscopic negative surgical margins; R1, microscopic positive surgical margins; RVR, rapid ventricular response; TOE, transoesophageal echocardiogram.

### 3.3 (Neo)‐adjuvant treatments

Preoperative systemic therapy was administered to three patients: one received immunotherapy prior to EPP, and two received neoadjuvant doxorubicin/ifosfamide (AI). One patient received postoperative vincristine/actinomycin/cyclophosphamide (VAC) for rhabdomyosarcoma. Two patients received adjuvant radiation (Table 1).

### 3.4 Disease outcomes

**FIGURE 1:**
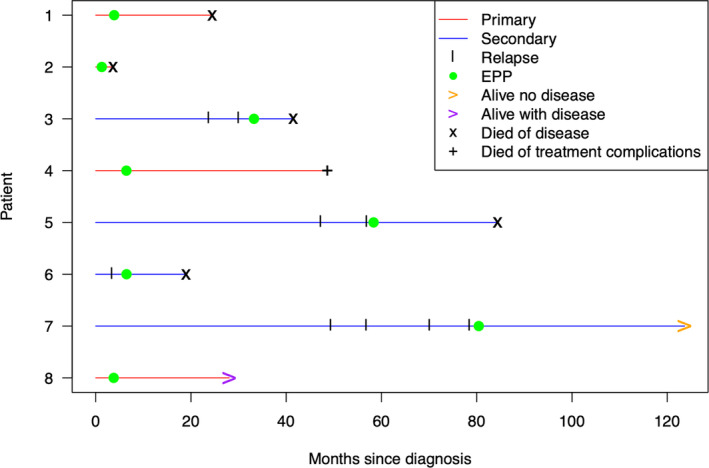
Swimmer plot of the timing of recurrence, timing of EPP, and overall survival for patients.

**FIGURE 2:**
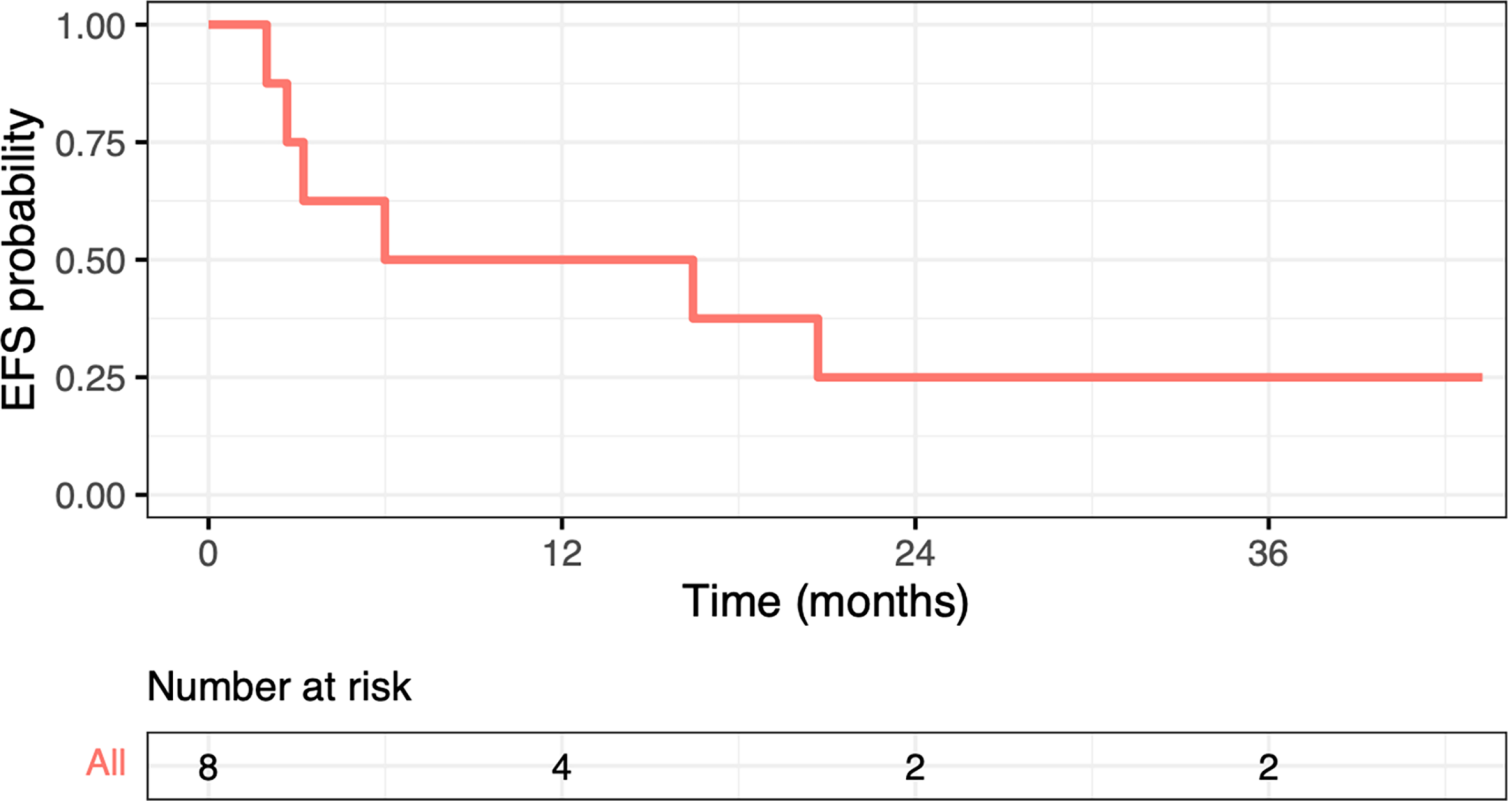
Event‐free survival of patients with pleural‐based sarcoma after extrapleural pneumonectomy. Kaplan–Meier estimates of event‐free survival probability of patients after extrapleural pneumonectomy are shown.

**FIGURE 3:**
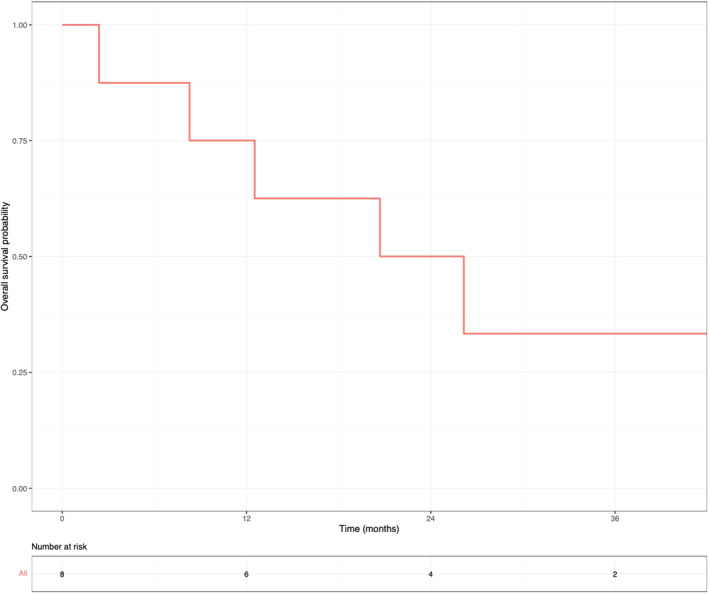
Overall survival of patients with pleural‐based sarcoma after extrapleural pneumonectomy. Kaplan–Meier estimates of overall survival probability of patients after extrapleural pneumonectomy are shown.

A swimmer plot of the timing of initial sarcoma diagnosis, disease recurrence, EPP, and the OS for each patient is shown in Figure 1. At a median follow‐up of 22.5 months, the median EFS after EPP was 6.0 months (Figure 2) and the median OS from EPP was 20.7 months, as calculated by the Kaplan–Meier method (Figure 3). Six patients (75%) had disease recurrence, including four distant and two local recurrences. At the last follow‐up, five patients (62.5%) died of progressive disease, and one was receiving palliative chemotherapy for local recurrence. Three patients, one with primary pleural‐based sarcoma and two with recurrent sarcoma (Patients 2, 3, and 6), had an early recurrence after EPP at 2.0, 2.7, and 3.2 months, respectively. Patient 2 underwent EPP for primary synovial sarcoma without perioperative treatment, rapidly recurred, and died before planned adjuvant radiation. Patient 3 had disease progression on immunotherapy for metastatic synovial sarcoma within 1 month prior to EPP; the patient also recurred rapidly after surgery and died. Patient 6 had a short DFI of 2.3 months after bleomycin/etoposide/cisplatin was completed for a non‐seminomatous germ cell tumor with subsequent transformation to rhabdomyosarcoma.

The patients who did not recur (Patients 4 and 7) or had an EFS of greater than 1 year (Patients 1 and 8), were observed to have either low‐intermediate grade histology or a demonstrated partial response to neoadjuvant chemotherapy.

Patient 4, who did not recur, received preoperative chemotherapy for a primary mediastinal synovial sarcoma and achieved a partial metabolic response on fluorodeoxyglucose (FDG)‐positron emission tomography (PET). The patient then underwent EPP and aortic grafting, followed by adjuvant radiation. Two years after the completion of treatment, the patient developed esophageal rupture and chronic aortic graft infection, which required multiple washouts for polymicrobial sepsis and long periods of hospital admission. The patient was disease‐free after follow‐up of 42.3 months but ultimately died of chronic sepsis.

Patient 7, who remained disease‐free 37.0 months after EPP, did not receive perioperative treatment. In contrast to the other patients in the case series, Patient 7 had a low to intermediate grade chondrosarcoma and multiple prior oligometastatic lung recurrences were treated with either resection or radiofrequency ablation. EPP was undertaken 6.7 years after the original diagnosis and 8.4 months after the most recent lung recurrence.

Two patients had prolonged EFS prior to disease recurrence (Patients 1 and 8). Patient 1 had a primary malignant solitary fibrous tumor and achieved a prolonged EFS of 16.4 months without pre or postoperative systemic treatment. The patient died of distant intra‐abdominal recurrence at 20.7 months. Of note, solitary fibrous tumor is considered a soft tissue tumor of intermediate grade malignant potential, which may explain the prolonged EFS despite lack of perioperative treatment.

Patient 8, with de novo metastatic undifferentiated pleomorphic sarcoma, had an EFS of 20.7 months. This patient received neoadjuvant chemotherapy with a partial metabolic response on FDG‐PET followed by resection and irradiation of a solitary brain metastasis before undergoing EPP. At last follow up of 23.0 months from EPP, Patient 8 was receiving palliative chemotherapy for inoperable local recurrence.

## 4 DISCUSSION

In our case series, EPP for adults with pleural‐based sarcoma was not curative for the majority of patients after a median follow‐up of 22.5 months. Only one patient (12.5%) remained alive without disease after follow‐up of 37 months, five patients (62.5%) died of direct disease‐related causes, one (12.5%) died of radiation complications, and one (12.5%) was receiving palliative chemotherapy for metastatic disease at last follow up. Our survival outcomes appear inferior to two case series published in 2022 and 2021 from Rodrigues et al.^7^ and Hameury et al.,^8^ respectively; however, there are striking differences between our patient population and those described in the earlier case series.

Rodrigues et al.^7^ reported the outcomes of 10 patients aged between 4 and 59 years (median 19.5 years) who underwent EPP, including eight with soft tissue sarcoma and one with bone sarcoma. After a median follow‐up of 29.2 months, five patients had no evidence of disease, three died of disease, and two died of treatment‐related complications, including heart failure due to constrictive pericarditis and radiation‐induced secondary sarcoma.

Hameury et al.^8^ described a case series of nine children aged between 9 and 17 years (median 15 years) with osteosarcoma (five cases), Ewing sarcoma (three cases), and undifferentiated sarcoma (one case). After a median follow‐up of 6.8 years, four patients were in complete remission, one patient was alive with local recurrence, and four died of metastatic disease.

Compared to Rodrigues et al. and Hameury et al., a much lower proportion of our cohort received perioperative and multimodal treatment, which may have contributed to inferior survival outcomes. In our cohort, only two of eight patients received neoadjuvant chemotherapy and two patients received RT, both given in the adjuvant setting. In contrast, nine of 10 patients received neoadjuvant chemotherapy (the exception was a patient with low‐grade fibromyxoid sarcoma) in the case series from Rodrigues et al., with 89% achieving a partial response, and all patients received RT either before and/or after EPP.^7^ Similarly, in Hameury et al., all nine patients received neoadjuvant chemotherapy and had achieved a good radiological response; six patients also received postoperative radiation.^8^ Our patient cohort was also much older, with a median age of 41 years compared to 19.5 and 15 years, respectively.

There is otherwise limited published literature on the outcomes of EPP for pleural‐based sarcoma in either children or adults, with only case reports available. Comparison with our cohort is challenging due to patient and disease heterogeneity. In a case report of two adults who underwent EPP for chondrosarcoma and hemangiopericytoma, both patients recurred at 14 and 43 months, respectively.^9^ In a case report, two children received EPP for sarcoma—one each of spindle cell sarcoma and inflammatory myofibroblastic tumor. The patients were disease free at 7 months and 3 years, respectively.^10^ In a case series of 13 patients with primary pulmonary sarcoma who underwent surgical resections of varying techniques and complexity, the three patients who underwent EPP died within 1 and 7 months after surgery; the histologies were one each of synovial sarcoma, malignant peripheral nerve sheath tumor and pleomorphic sarcoma.^3^ In general, the histological subtypes and the perioperative treatments reported in the literature are very heterogenous, making comparisons with our cohort difficult.

Regarding surgical mortality and morbidity, our findings of three patients (37.5%) with early postoperative complications and no deaths by 30‐ and 90‐days are comparable to those reported by Rodrigues et al. and Hameury et al. Rodrigues et al. described one of 10 patients developed an early complication of empyema that necessitated wound washout and one patient of ten patients who developed a late complication of fatal pericarditis and died 7 months post‐surgery.^7^ In Hameury et al., three of nine patients had early postoperative complications including one case of pulmonary infection, one case of postoperative bleeding requiring surgery and one case of cardiac tamponade managed with an emergency pericardial window. Two patients developed late postoperative complications, one with ulcerated esophagitis due to hiatal hernia and one with feeding problems, which resolved after gastrostomy. The median hospital length of stay in our cohort was 14 days (range 9–45), comparable to that reported in Hameury et al. of 14.5 days (range 8–24).^8^

Furthermore, the surgical complication rates, the median operative time, and the median length of hospital study for patients in our cohort compares favorably with results of EPP performed for malignant pleural mesothelioma. A systematic review of 2462 patients from 34 studies who underwent EPP for mesothelioma found the overall perioperative mortality rates ranged from 0% to 11.8% and the morbidity rate ranged from 22% to 82%^11^; the median operative time ranged from 3.25 to 6.5 hours, and the median length of hospital stay ranged from 8 to 43 days. These results are similar to our perioperative complication rate of 37.5%, perioperative mortality rate of zero, median surgical operative time of 4 hours and median hospital length of stay of 14 days.

The variable survival outcomes of patients in our study confirms the importance of patient selection for EPP. Previous publications have proposed the following criteria regarding patient selection for EPP^3^, ^7^, ^8^: (1) recommendation by consensus opinions of multidisciplinary tumor board discussion; (2) pleural lesions limited to one hemithorax; (3) cases where there is no other alternative apart from palliative treatment; (4) good performance status; and (5) “sufficient” response to preoperative chemotherapy. Several authors have discussed the importance of EPP being offered as part of multimodal treatment in conjunction with chemotherapy and/or radiation and have cautioned that surgery alone may be insufficient to provide long‐term disease control. However, what satisfies a “sufficient” response is unclear, but based on our series, at least a partial radiological response appears to be associated with superior outcomes.

The patients who underwent EPP for pleural‐based sarcoma in our study all met selection criteria 1–4. The small numbers in our case series preclude formal statistical analysis of the impact of clinicopathological characteristics on EFS and OS, however, the characteristics of patients with the longest survival were: low‐intermediate grade histology, a DFI of more than 6 months from the completion of treatment for the last incidence of disease to EPP, and a demonstrated response to preoperative chemotherapy. In contrast, patients with DFI less than 6 months or disease progression on systemic treatment shortly before surgery did not benefit from EPP, indicating an inherently aggressive tumor biology.

To our knowledge, this is the largest case series of outcomes of EPP undertaken in adults with pleural‐based sarcomas. The limitations of this study include its retrospective nature, small sample size, and the inclusion of a variety of histological subtypes with varied biological behavior and treatment sensitivities, which undoubtedly influence disease outcomes. However, gathering high level evidence is challenging given the rarity of patients with pleural‐based sarcoma, the limited number of patients with this diagnosis suitable for EPP and the small number of high‐volume centers worldwide that are able to offer this complex surgical procedure. Ultimately, international collaboration to identify a larger case series and systematic reviews will be required to deduce which patients will most likely benefit from EPP and the optimal patient selection criteria.

## 5 CONCLUSIONS

In conclusion, EPP is rarely curative in adults with pleural‐based sarcoma, however, it can be performed with acceptable surgical risks when performed by an experienced thoracic team in specialized centers. Careful patient selection is critical given the potential for significant morbidity and poor survival outcomes associated with EPP. Building on previously reported selection criteria, we suggest patients being considered for EPP should receive preoperative treatment with chemotherapy and demonstrate a confirmed response prior to proceeding to EPP. In addition, selection criteria for EPP may also include patients with low‐intermediate grade tumor histology and a longer disease‐free interval (more than 6 months) from the last incidence of tumor. Patients being considered for EPP should receive comprehensive counseling regarding the intent of EPP, whether curative or life‐prolonging by controlling local disease burden and the likelihood of achieving this aim, the available evidence on long‐term outcomes, and the potential morbidity associated with EPP. Formal statistical analysis of the impact of clinicopathological characteristics on EFS and OS could be explored in future studies if an adequate number of patients undergoing EPP for pleural‐based sarcoma can be identified; this would allow confirmation of the patient selection criteria as discussed and identify other potential refinements to patient selection criteria.

## AUTHOR CONTRIBUTIONS

**Betty Y. Zhang:** Data curation (lead); formal analysis (lead); writing – original draft (lead); writing – review and editing (lead). **Ashley R. Wilson‐Smith:** Data curation (supporting); formal analysis (supporting); writing – original draft (supporting); writing – review and editing (supporting). **Elizabeth A. Connolly:** Writing – original draft (supporting); writing – review and editing (supporting). **Madeleine C. Strach:** Writing – original draft (supporting); writing – review and editing (supporting). **Nathan Ussher:** Data curation (supporting); formal analysis (supporting). **Tristan Yan:** Conceptualization (equal); investigation (lead); supervision (supporting). **Vivek A. Bhadri:** Conceptualization (equal); funding acquisition; supervision (lead); writing – review and editing (supporting).

## CONFLICT OF INTEREST STATEMENT

The authors have stated explicitly that there are no conflicts of interest in connection with this article.

## ETHICS STATEMENT

Ethics approval for this study was granted by the Royal Prince Alfred Hospital Research Governance Officer (Approval number: X19‐0246 and 2019/STE14629).

## Data Availability

The data that support the findings of this study are available from the corresponding author upon reasonable request.
